# The friends and family test: a qualitative study of concerns that influence the willingness of English National Health Service staff to recommend their organisation

**DOI:** 10.1177/0141076814532392

**Published:** 2014-08

**Authors:** Mary Dixon-Woods, Joel T Minion, Lorna McKee, Janet Willars, Graham Martin

**Affiliations:** 1 SAPPHIRE group, Department of Health Sciences, University of Leicester, Leicester LE1 6TP, UK; 2 Data to Knowledge group, School of Social and Community Medicine, University of Bristol, Bristol BS8 2BN, UK; 3 Health Services Research Unit, University of Aberdeen, Aberdeen AB25 2ZD, UK

**Keywords:** quality of care, net promoter, staff concerns, soft intelligence, National Health Service

## Abstract

**Objectives:**

The views of practitioners at the ‘sharp end’ of care provision are increasingly recognised as important indicators of quality of care. The National Health Service (NHS) Staff Survey in England has quantified employees' views on how far they would be happy with the standard of care provided by their organisation if a friend or family member needed treatment. We aimed to characterise the concerns that might affect the willingness of staff to recommend their own organisations.

**Design:**

Qualitative study involving semi-structured interviews. Data analysis based on the constant comparative method.

**Participants:**

Members of clinical and managerial staff in four NHS organisations (*n* = 70), and senior stakeholders across the NHS including clinicians, managers and others with a strategic or senior-level perspective (*n* = 98).

**Setting:**

One hundred and sixty-eight interviews were conducted: 70 in four case study organisations and 98 across the wider English NHS.

**Main outcome measures:**

Not applicable.

**Results:**

Asking study participants the ‘if a friend…’ question offered insider views on the quality of care. Some staff had no concerns, but others, identified significant problems with consistency, reliability and behaviour of staff. Participants identified reasons for poor care that included inadequate organisational systems; structural problems of understaffing and under-resourcing; weaknesses in professional cultures and professional competence and failure to deal with problems such as unacceptable conduct. Participants emphasised that staff were not always able to deliver high-quality care because they worked in difficult conditions.

**Conclusions:**

Asking staff to give accounts of their willingness to recommend their organisation to family and friends elicits important insights into quality and safety of care. Such accounts might be able to provide warning signs that could signal organisational decline and avert healthcare scandals, but use outside a research context requires further evaluation.

## Introduction

The views of practitioners at the ‘sharp end’^1^ of care provision are increasingly recognised as important indicators of quality of care.^2–7^ Studies have shown associations between measures of staff morale and satisfaction and clinical outcomes, patient safety and patient satisfaction.^8–11^ Direction of causality in such studies remains difficult to establish, but the views of staff are increasingly seen as one important signal of organisational culture relating to patient safety and quality of care. Inquiries led by Sir Robert Francis into failures of care at Mid Staffordshire National Health Service (NHS) Trust in England, for example, identified staff concerns about care quality in the hospital's emergency department as a warning sign that went unheeded.^12^ The public inquiry suggested that staff feedback on quality of care be ‘not only encouraged but insisted upon’ in healthcare organisations.^12^

One important way of obtaining data on the views of those at the sharp end is through the use of a so-called net promoter question, which assesses how far staff are prepared to recommend their own organisation to others.^13^ A version of this question has been asked on the NHS National Staff Survey in England annually since 2009: staff across organisations have been asked to rate on an ordinal scale the extent to which they agree with the statement ‘If a friend or relative needed treatment I would be happy with the standard of care provided by the organisation.’^14^ Some evidence suggests a correlation (albeit weak) between the proportion of staff who give a positive response (i.e. ‘agree’ or ‘strongly agree’) and hospital standardised mortality ratios.^15^ In 2013, the most recent year for which data are available, the proportion of staff who gave a positive response was in the range of 39.5–94%, with a mean of 67%.^16^ This suggests that while on balance many staff are supportive of the care provided, substantial variability between different organisations exists.

Much less clear, however, is what explains staff's willingness to recommend their organisation in response to the ‘if a friend…’ question, and in particular the kinds of concerns that might affect the extent to which they feel they can endorse their organisation. In this article, we present an analysis of qualitative interviews that helps to address this deficit in current knowledge.

## Methods

We conducted interviews with clinicians, managers and others directly involved in the NHS as part of a wider study involving interviews and organisational case studies of culture and behaviour in relation to quality and safety in the English NHS that was conducted over the period 2010–2012.^17^ The topic guides for interviews included the question ‘if a friend or relative were treated in your organisation, what would concern you most?’ Responses to this question were followed up by probing prompts to explore participants' initial responses further.

Our sampling strategy for both the case studies and the telephone interviews was designed not to achieve statistical representativeness but to obtain a range of views from a breadth of individuals involved in the management, direction and delivery of care about the current state of quality and safety in the NHS. All interviews were audio-recorded and then transcribed in full.

The ‘if a friend…’ question was asked in 70 face-to-face interviews with staff conducted in four NHS case study organisations which had been involved in the wider project.^17^ In one of these four organisations, the percentage of staff who had indicated positive agreement on the NHS Staff Survey that they would be happy for a friend or family member to be treated in their own organisation was in the range 50–60%, in the second it was 60–70%, and in the third it was 70–80%. These sites thus represented a range of responses but did not include any organisations in the bottom quartile of performance on this measure. Data were not available for the fourth site, which was a primary care organisation that did not participate in the annual survey.

The ‘if a friend…’ question was also asked in 98 telephone interviews with senior stakeholders across the NHS^18^ comprising clinicians, managers and others with a strategic or senior-level perspective on the NHS and based in settings including acute, primary care and mental health. These individuals were drawn from 76 different organisations, of which 51 were involved in provision of direct patient care; the remainder were in a variety of roles including commissioning (e.g. primary care trusts) and system oversight (e.g. strategic health authorities). Many participants had dual roles both in providing care and management (e.g. clinical directors), making it difficult to distinguish precisely between those at the sharp end and those at the strategic end. The 98 stakeholders were drawn from a larger sample of 107;^1^ the question was not asked in interviews where stakeholders did not have direct roles in healthcare provision or oversight.

Data analysis was based on the constant comparative method.^19^ The entire dataset of 168 interviews was analysed together. Since our aim was to characterise the nature of staff concerns, we did not attempt to treat the case study organisations as analytic categories. We did not relate statements by participants to scores on the Staff Survey, nor did we compare responses in relation to professional background or level of seniority. The analytic process involved reading and rereading transcripts,^34^ then, with the assistance of Nvivo software, coding excerpts of text according to themes emerging from the data themselves and from the prior sensitising concepts,^20^ including, for example, the London protocol for systems analysis of clinical incidents. Subsequently, codes were refined through development, discarding, merging and disaggregating, giving rise to a final coding framework that was validated by GPM.

We recognise that positive staff insights can provide learning opportunities and insights as to successful care. However, for the purposes of this paper, we focus primarily on those who expressed concerns rather than on those who expressed complete confidence in their organisations. Such candid reflections have often been poorly captured, synthesised or codified.

## Findings

Interviews were carried out with 168 individuals representing diverse occupations in the NHS, including healthcare assistants, doctors, nurses, managers, senior executives and other professional groups (Table 1). Recruitment from the ‘sharp end’, where staff were involved in direct patient care, and the ‘blunt end’^2^ of organisational policy-making and decision-making (e.g. those in executive or board roles) was variable across the case studies for reasons specific to each site (including research governance permissions and ability to access clinical areas). In keeping with the sampling strategy, the stakeholder sample was weighted towards those in senior positions.

**Table 1.** Breakdown of interviewees by sub-study.

	Case 1	Case 2	Case 3	Case 4	Case totals	Stakeholders	Total
Sharp-end interviews	17	4	0	12	33	98	
Blunt-end interviews	7	0	20	10	37		
Total	24	4	20	22	70	98	168

Around one-quarter (45/168) of all participants reported that they would be happy to recommend their organisation to family and friends and suggested that they would have no worries about the quality of care. While these positive responses are important, they will not be examined in any depth here given our focus on identifying staff concerns.

I know we get it right nearly every time. (Stakeholder 3, manager)

I think I'd honestly be quite, quite proud to recommend the organisation to a close friend. […] I wouldn't have any qualms at all about friends or relatives using our service. (Case study participant 42, director)

At the other extreme, 12 participants reported that they would be concerned by ‘everything’.

Everything would worry me. I mean I can honestly say that because my father has recently been in hospital and I watched him like a hawk. (Stakeholder 63, clinician)

In between these two ends of the spectrum (no concerns to multiple concerns), most participants indicated that it was not so much that they could not identify anything good about their organisation but that they were anxious about the reliability and variability of quality of care across their organisations: they could not be sure that care would be consistently good always and everywhere. They emphasised that ‘bright spots’ of high-quality care could be found throughout the NHS both across and within trusts but feared that ‘dark spots’ of care also existed.^3^

I've worked in 15 different trusts throughout my whole training and I think this is probably the only place that I'd want them to be treated. (Case study participant 16, consultant anaesthetist)

In some areas I would have no worries at all, in others […] I know which wards are doing better and which wards are not […] if my friend was on one of the wards that was having difficulties, I might worry about drugs being given regularly, observations being taken properly, rescuing them if they started to deteriorate, those sorts of things. Whereas on another ward I would not have any concerns about that at all. (Stakeholder 2, chief executive)

Participants often gave detailed accounts of the reasons why they might be concerned about recommending their organisation to a friend or relative. These could be categorised as relating to structural, management and systems issues; culture and behaviour, including concerns relating to the conduct of specific individuals; and lack of consistency of care.

### Structural, management and systems issues

Structural issues relating to resources, both finance and levels of staffing and the availability of the right skills, were identified as important influences on quality and safety of care across our interviews:

It's all demand all the time, and much more turnover of patients, and I don't know that I could put my hand on my heart—and not just in my own hospital, because I've been round several hospitals—and think that I could confidently leave anybody there and be assured that they were 100 per cent of the time going to get good care. (Stakeholder 31, assistant director of nursing)

Sometimes the level of cover is stretched very thinly and is very junior. (Stakeholder 6, consultant microbiologist)

Just not enough staff in there, they have a very difficult time retaining their nurses because it's such an intense, busy, stressful environment. (Case study participant 2, unit manager)

A substantial number of concerns (expressed by 39 participants) focused on the extent to which organisational systems and managerial goals supported and enhanced, rather than impeded, the quality of care.

It's hard to get problems properly prioritised and I think that is a safety and quality problem. […] If you get referred for a condition that doesn't appear to be cancer you're liable to be put on a very long waiting list to see someone in a clinic and it can be very, very difficult to bring to the clinician's attention the fact that there are features about this case that make it a priority that needs to be seen urgently. (Stakeholder 85, consultant surgeon)

I'd be embarrassed if a relative of mine came in as a general surgical emergency, because sometimes they have to wait for hours to be seen. […] There have been instances where patients have suffered because they haven't been seen quick enough. It's shameful really. (Case study participant 5, senior administrator)

Participants drew attention to how national targets and priorities were translated into local policies in ways that made it difficult to prioritise higher risk patients, but they also emphasised how problems in local-level systems, processes and protocols could interfere with high-quality care. In 53 accounts, participants suggested that quality and safety lost out in competition with priorities such as finance:

In the wider trust I know that the CQC [regulator's report] wasn't good, that the mandatory training wasn't good. That the staffing levels aren't necessarily great. And that there's there appears to be a very big focus on finance. So all of those would be my concern I guess. (Case study participant 13, trainer)

The occupancy rate is just too high I think. I don't think staff get a break at all. I think we are at 98% occupancy a lot of the time, it is meant to be 80 but it is at a minimum of 90 if not 95. Just too many patients, too many people coming in. There is financial pressure to close wards so you close wards and that means you have got no beds. Like we have got no beds today at all. (Stakeholder 50, deputy director of nursing)

Boundaries between different systems of health and social care were seen as an area particularly vulnerable to quality and safety problems; challenges of coordination and communication of relevant information were recurrent themes in many accounts.

The [discharge] information may or may not include anything to do with the reasons why there are changes and it may or may not include something about for example the dose of these medicines, let's say somebody's heart failure might need to be increased gradually over the next 6 weeks or something. And you know the GP is not a mind reader, so can only work with the information that they've got. (Stakeholder 71, senior pharmacist)

### Culture, competence, behaviour and conduct

Forty-six participants highlighted concerns directly associated with the quality of clinical care, including competence of staff in diagnosis, selection of appropriate therapy and administration of interventions; 24 emphasised concerns about the behaviour and conduct of staff. Particular concern focused on the competence of newly qualified doctors, the consistency of the quality of nursing and the abilities of team leaders.

I am ashamed to say that some of my colleagues can treat people like that. I got really upset and I just said, ‘What would it be if it was your father or your mother or whatever sat in that?’ I said. My dad is blind as well, so he doesn't see a lot of things, they were leaving his food there and they didn't feed him, he couldn't feed himself so we were coming in and his food was still there on the table. That is not nursing care. (Case study participant 41, operating department practitioner)

‘Culture’ was a recurring theme in how participants accounted for these problems in quality and safety, but it was seen not necessarily as a homogeneous organisational property. Rather, it was seen to vary between different clinical areas and across professional groups and to be influenced by team, structural and systems issues (such as those considered above, including understaffing and training).

I think I would be most worried about manner and attitude. I think that is something we need to focus on here. […] Just people being rude, the way that some staff talk to patients and just generally the way they seem to be quite dismissive of certain people. (Stakeholder 39, ward manager)

If nurses don't know their patients and aren't caring for them and aren't talking to their relatives, and aren't involved more in their day-to-day wellbeing, I think that's, that's a great loss. (Stakeholder 1, consultant histopathologist)

The manager of that [district nursing team] was not a nurse. She never knew her nurses. She never knew the capability or the standard of care they were delivering. (Stakeholder 57, clinical services manager)

Many accounts stressed that problems of behaviour, communication and culture – in particular compassion and care for patients – were influenced by notions of professionalism but were also powerfully shaped by the conditions in which people were asked to work. Problems with caring were mostly seen not as simple individual deficits or features of particular professional groups, but as arising in contexts where staff were harassed and over-stretched by too many competing demands and priorities and/or lacked effective leadership.

I know that on a certain ward generally I would be concerned because of staff attitude […] We have issues with a lot of things, like a lot of trusts, but it's variable depending which ward, and that's very much about the leadership on the ward as well. (Stakeholder 25, senior nurse)

It's not about the food or the doctors or the nurses, it's about people's attitude. General actually wanting to care. It all seems so quick. […] It all seems so boom boom boom, you're in here, we'll fix you, we'll send you home again. […] I think it's a general thing where everybody is understaffed, overworked, underpaid, you know, the same old. But at the moment it's coming to fruition because we have different government standards, we're having so many cuts and people are just exhausted. And they forget that bit. (Stakeholder 81, clinical nurse specialist)

Nevertheless, some participants did identify that their concerns were founded not just on generalised worries about culture, systems and priorities but on the conduct and competencies of individual practitioners:

There are different consultants who have different skill sets, and we all know who you would let treat your wife and we all know who you wouldn't. (Case study participant 16, consultant anaesthetist)

…in any hospital what would worry me is, was she lucky enough to be there on a day when she got a great doctor and a great nurse? (Stakeholder 31, assistant director of nursing)

### Variability in care

Problems of quality and safety were perceived to affect some patient groups more than others. Older people were seen as particularly vulnerable.

If I had a very elderly relative, I would not be confident that they were always going to be helped to eat or they would get enough food. (Stakeholder 69, gastroenterologist)

Some participants suggested that while the NHS was very effective at dealing with emergency situations, it was more likely to fall short in terms of patient experience, quality and safety of care for patients with multiple morbidities and longer term, complex needs. Relatedly, continuity of care was identified as a problem by 19 participants:

I think if you are really [acutely] ill you are fine in the NHS…. I think where it falls apart is if you have got a long-term condition that is not well managed. (Stakeholder 34, assistant director of nursing)

[…] we have a fairly high turnover of clinical staff [in primary care] and that's one of the things we are constantly trying to address, probably the one area of weakness that we do have. As such, yeah, it would be the one thing I would highlight to a friend if they had a chronic medical condition. (Case study partcipant 49, director)

Problems of quality and safety were also perceived to vary according to time. Seasonal variation (‘winter pressures’) and out-of-hours care at weekends and overnight were identified as particular pressure points:

Over winter it's been a very difficult period and I think that is because we have struggled to manage the beds. We have struggled to provide the capacity and I think what is worrying is when patients then don't end up in the right specialty. (Case study participant 9, patient services manager)

The only thing that would probably have some concern is the out-of-hours cover. […] it's not the care I'd get on the ward but if I became unwell. It's the support I would get to look after me if I became more and more unwell. (Case study participant 18, deputy chief executive)

The riskiest situations for quality and safety of care were seen by staff to lie in the ‘perfect storms’ that occurred when high-dependency patients with complex needs and overlapping or poorly demarcated professional responsibilities interacted with weak systems, poor transfers or handoffs, understaffing and professional cultures that neglected the holistic care of patients:

Nursing care there, but also a lot of other mitigating factors that [the patient] has been bounced around three wards, because nobody wanted to actually take responsibility [for] his care […]. So therefore his care suffers because nobody knows what to do with him. (Case study participant 10, operating department practitioner)

## Discussion

The extent to which staff are prepared to recommend their own organisation to others is increasingly promoted as valuable indicator of quality and patient safety in healthcare organisations.^21,22^ Measures on the NHS Staff Survey and others using similar ‘net promoter’-style approaches might be regarded as a form of (relatively) hard data. In identifying what lies behind the scores obtained on survey scores and what to do in response, softer intelligence about the nature of staff concerns is, as Sir Robert Francis identified in his report, also necessary.^23^ Asking the ‘if a friend…’ question in the context of an interview allowed participants to offer their insider views on the delivery of care in organisations, the kinds of problems that arise, the patient groups most vulnerable to poor-quality care and the ‘pinch points’ where weaknesses in healthcare systems might result in suboptimal care for patients.

Our study suggests that many participants' reluctance wholeheartedly to endorse their organisation may derive mostly from perceptions of inconsistency, specifically in relation to quality of clinical care across clinical areas, adequacy of systems and management, and the culture, communication and behaviour of caregiving staff. Providing consistently high-quality care to patients with complex needs, older patients and those with multiple co-morbidities or long-term conditions was seen as a particular challenge. Resource pressures, including working conditions and inadequate staffing levels, were seen as strongly influencing attitudes and behaviours. Despite the emphasis on structural and systems factors, participants did not exclude lapses in individual professional conduct as the source of some difficulties in assuring quality of care.^24^

Though some participants (7%) said they had grave concerns about too many aspects of their organisation to enumerate, a significant proportion (27%) of those we interviewed did express unreserved willingness to recommend that a friend or family be treated in their own organisation. In future studies, it may be important to reflect on what can be learned from this group and to compare and contrast feedback from within organisational case studies as well as between organisations to try to decipher what seems to lead to such positive endorsements and high self-reports.

Though our study is large by the standards of much qualitative research, and had the advantage of sampling across the English NHS, it has some limitations. It may, particularly in the stakeholder component of the study, have over-sampled the views of senior-level rather than frontline staff. Though our sample did include primary care, community care and mental health perspectives, views from these sectors were under-represented and our analysis may have privileged concerns relating to acute care. We did not attempt to distinguish the views of participants according to professional group or discipline, nor did we compare the accounts from different staff groups from within the same organisation, nor undertake a cross-case analysis. Though some research has reported differences between the perceptions of professional groups and patients about the quality of care,^25–27^ others have suggested systematic differences in staff views on quality between organisations and fields of care.^28,29^ We were unable to compare the accounts of participants against other measures of quality and patient safety, nor was it possible to verify what they said. Nonetheless, our findings offer important insights into the views of NHS employees that might otherwise be difficult to access about perceived limitations in local care provision.

The concerns identified by staff were diverse; different responses may therefore be required to address them. Some concerns – for example, those relating to the competence or conduct of individual staff – might be managed through individual-level interventions such as training, professional support or performance management and/or disciplinary action. But detecting such concerns and acting on them effectively may require a sophisticated organisational infrastructure, including high-quality human resource processes.^30^ Concerns relating to weak organisational systems and processes need to be addressed though sustained efforts and the right kinds of skills and through building mechanisms to ensure that local leaders are alerted and attentive to these concerns as well has having the ability to address them effectively.^31^ Structural-level questions of staffing levels and skill-mix are likely to require attention to the scientific evidence and sensitivity to local context.^32^ Problems in professional cultures identified by some participants imply the need for wide-ranging changes at the levels of socialisation, recruitment and career incentives.^12,33^ All of these efforts will require high-quality leadership, engagement with staff and excellent management practices.

Our findings suggest that going beyond survey responses to ask staff directly about their reflections on recommending their organisation to a friend or family member might offer an untapped source of intelligence for healthcare organisations. Accounts from staff such as these could provide early warnings of the kind that could be so useful in detecting and dealing with suboptimal care.^12^ Some cautions are necessary in assuming that re-tooling our methods for management purposes would be straightforward, however. The research setting for this study – with anonymisation, confidentiality and a clearly declared (non-managerial) purpose – is likely in part responsible for the frank, and sometimes startlingly candid, responses we gained as well as the absence of unconstructive self-deprecation or self-congratulation. We therefore have some confidence that our findings reflect shared concerns of many NHS staff. But asking similar questions towards a different end – gathering intelligence by senior staff, for example, or comparing quality across organisations – would require further evaluation and development.

## Conclusion

Staff views of their own organisations and the development of methods to collect local intelligence on performance, culture, communication and behaviour can provide useful and actionable insights into quality and patient safety in healthcare settings. Qualitative interviews allow staff to provide detailed accounts of the nature of quality and safety problems and their causes but will require further evaluation for use outside a research setting.

## Declarations

### Guarantor

MD-W

### Provenance

Not commissioned; peer-reviewed by Susan Burnett
